# Potassium Permanganate in Amenorrhœa

**Published:** 1887-03

**Authors:** 


POTASSIUM PERMANGANATE IN AMENORRHŒA.—Dr. Marshall, of San Francisco, after employing this drug in fifty cases of amenorrhœa, has arrived at the following conclusions:

1. The permanganate acts satisfactorily in about seventy per cent. of "selected cases."

2. It should be administered one or two hours *after* eating.

The disagreeable action on the stomach may be relieved by combining it with the following:

| | |
|---|---|
| Oxalate of cerium, - - - | 1 grain. |
| Hydrochlorate of cocaine, - - - | $\frac{1}{6}$ grain. |
| Subnitrate of bismuth, - - - | 5 grains. |
| Powdered ipecac, - - - - | $\frac{1}{60}$ grain. |

The writer also states that this drug has a marked tonic effect and generally causes mental exhilaration.—*Canadian Practitioner.*

